# A network pharmacology approach to decipher the mechanism of total flavonoids from *Dracocephalum Moldavica* L. in the treatment of cardiovascular diseases

**DOI:** 10.1186/s12906-023-04316-x

**Published:** 2024-01-02

**Authors:** Rui-fang Zheng, Kaderyea Kader, Di-wei Liu, Wen-ling Su, Lei Xu, Yuan-yuan Jin, Jian-guo Xing

**Affiliations:** 1 Xinjiang Key Laboratory of Uygur Medical Research, Xinjiang Institute of Materia Medica, Urumqi, 830004 China; 2 Department of Clinical Pharmacy, School of Preclinical Medicine and Clinical Pharmacy, China Pharmaceutical University, Nanjing, 211198 Jiangsu China; 3 Institute of Medicinal Biotechnology, Dongcheng District, Chinese Academy of Medical Sciences, No. 1 Tiantanxili, Beijing, 100050 China

**Keywords:** Total Flavonoids from *Dracocephalum Moldavica L*, Cardiovascular disease, Network pharmacology, Pharmacological evaluation, Recombinant NADPH oxidase 4

## Abstract

**Aim of the study:**

Cardiovascular disease (CVD) seriously endangers human health and is characterized by high mortality and disability. The effectiveness of *Dracocephalum moldavica* L. in the treatment of CVD has been proven by clinical practice. However, the mechanism by which DML can treat CVD has not been systematically determined.

**Materials and methods:**

The active compounds in DML were screened by literature mining and pharmacokinetic analysis. Cytoscape software was used to construct the target-disease interaction network of DML in the treatment of CVD. Gene ontology and signalling pathway enrichment analyses were performed. The key target pathway network of DML compounds was constructed and verified by pharmacological experiments in vitro. A hydrogen glucose deprivation/reoxygenation model was established in H9c2 cells using hypoxia and glucose deprivation for 9 h combined with reoxygenation for 2 h. The model simulated myocardial ischaemic reperfusion injury to investigate the effects of total flavonoids of Cymbidium on cell viability, myocardial injury markers, oxidative stress levels, and reactive oxygen radical levels. Western blot analysis was used to examine NOX-4, Bcl-2/Bax, and PGC-1α protein expression.

**Results:**

Twenty-seven active components were screened, and 59 potential drug targets for the treatment of CVD were obtained. Through the compound-target interaction network and the target-disease interaction network, the key targets and key signalling pathways, such as NOX-4, Bcl-2/Bax and PGC-1α, were obtained. TFDM significantly decreased LDH and MDA levels and the production of ROS and increased SOD activity levels in the context of OGD/R injury. Further studies indicated that NOX-4 and Bax protein levels and the p-P38 MAPK/P38 MAPK andp-Erk1/2/Erk1/2 ratios were suppressed by TFDM. The protein expression of Bcl-2 and PGC-1α was increased by TFDM.

**Conclusions:**

Our results showed that DML had multicomponent, multitarget and multichannel characteristics in the treatment of CVD. The mechanism may be associated with the following signalling pathways: 1) the NOX-4/ROS/p38 MAPK signalling pathway, which inhibits inflammation and reactive oxygen species (ROS) production, and 2) the Bcl-2/Bax and AMPK/SIRT1/PGC-1α signalling pathways, which inhibit apoptosis.

**Supplementary Information:**

The online version contains supplementary material available at 10.1186/s12906-023-04316-x.

## Introduction

Cardiovascular disease (CVD) is a leading cause of death and disability worldwide, and its incidence is increasing in low- and middle-income countries (LMICs) [1]. The number of deaths caused by CVD increased by 12.5% from 2005 to 2015 and reached 17.8 million worldwide. The ranking of ischaemic heart disease (IHD) and stroke mortality rose from fourth and fifth place in 1990 to first and second place in 2015 [2]. CVD has become the “number one killer” that seriously endangers human health and life. Conventional therapeutic drugs for cardiovascular diseases include antihypertensive drugs such as diuretics, angiotensin converting enzyme (ACE) inhibitors, beta blockers, blood thinning drugs (to reduce platelet aggregation), cholesterol lowering drugs and/or antiarrhythmic drugs. However, these drugs also have certain side effects, such as fatigue, shortness of breath, headache, and dizziness [3], and rhabdomyolysis and hypolipidaemic drugs can cause liver disease. Traditional Chinese medicine (TCM) has a rich history in the treatment of CVD. TCM contains multiple compounds that have multitarget effects, which are more effective than single drug treatments, especially for the treatment of complex chronic diseases such as schizophrenia, depression, diabetes and cardiovascular disease [4].

In recent years, network pharmacology has been used to examine traditional Chinese medicine with modern biochemical tools to improve or increase diagnostic descriptions, and the use of Western concepts based on biochemistry, pathways or regulatory processes have been used to explain TCM theory [5]. The combination of systems biology and pharmacology provides a new network/path analysis method for the treatment of complex diseases by traditional Chinese medicine by using pharmacokinetic evaluation and targeted prediction [6, 7]. As a comprehensive method for systematically investigating and interpreting TCM and its molecular mechanism, TCM network pharmacology represents a complex biological system that breaks traditional analytical chemistry and pharmacology techniques to effectively establish a compound-protein/gene-disease network and examine the regulation of small molecules in a high-throughput manner. Therefore, TCM network pharmacology has become an effective tool for analysing drug combinations to provide new ideas and means to study Chinese medicines with multicomponent and multitarget characteristics.

*Dracocephalum Moldavica L*. (DML) is a traditional Uighur medicine with the Uyghur name BadiRanjibuya, which is used in Xinjiang and included in the Uighur classical medical book “Aricanon”. More than 800 years of clinical practice has proven that DML is effective in the treatment of CVD. There are nine prescriptions for the treatment of CVD in the “Medical Standards of the Ministry of Health of the People’s Republic of China Uyghur Medicine Volume”, and three contain DML [8]. For more than ten years, our team has been committed to performing research on DML in the treatment of CVD. Pharmacological experiments have shown that DML has antioxidative effects, scavenges oxygen free radicals and provides myocardial protection [9]. DML is a natural medicine with great potential for research and development. However, most studies have focused on pharmacodynamics, and there have not been any systematic analyses of the mechanism by which DML treats CVD, such as the targets, molecular mechanisms and interactions between components of DML, which need to be further studied.

In the present study, we used computational tools and resources to investigate the pharmacological network of DML in CVD to predict the bioactive compounds in DML, protein targets and pathways. We also performed in vitro experiments to validate the underlying mechanism by which DML affects CVD, as predicted by a network pharmacology approach. To the best of our knowledge, this is the first time that a potential mechanism for the treatment of CVD by DML has been studied by network pharmacology and in vitro experimental verification.

## Materials and methods

### Screening of active compounds in DML

All candidate components of DML were collected from the traditional Chinese Medicine System Pharmacology (TCMSP) database (http://tcmspw.com/) [10] and related reports. In addition, the bioactive ingredients that contribute to its therapeutic effect were screened using the pharmacokinetic parameters ADME, such as human oral bioavailability (OB), drug likeness (DL), Caco-2 permeability (Caco-2), half-life (t1/2), and blood‒brain barrier (BBB) permeability.

OB is closely related to drug efficacy and one of the most important pharmacokinetic parameters in ADME (absorption, distribution, metabolism, excretion) [11], representing the percentage of a drug effect that a unit of an oral dose can produce. In this study, the OBioavail1.1 predictive model and the IntegOB predictive model were used to accurately calculate the OB of the compound, and the candidate components were required to meet the following parameters: oral bioavailability (OB) ≥ 20%.

DL refers to the property of a drug similar to known drugs in previous studies, and both agents have similar or identical physicochemical properties and functional groups. DL is a concept generated during the development of drugs and oral drug production. DL also reflects the structural characteristics, physicochemical properties and a series of parameters of drug molecules in vivo. Therefore, DL is often used as an important criterion for judging whether a compound can be developed as a new drug. In early screening, this characteristic plays an important role in improving the selection probability of candidate drugs. In this study, the DL of each molecule was predicted, and effective molecules with the possibility of drug formation were screened using the Tanimoto coefficient (Eq. (1)).

1 $${\text{T}}\left({\text{X}},{\text{Y}}\right)=\frac{x\cdot y}{{\left|x\right|}^{2}+{\left|y\right|}^{2}-x\cdot y}$$

In the formula, X represents the descriptor of each candidate component, and Y represents the average value of the descriptors of all the drugs present in the DrugBank database (http://www.drugbank.ca). Since the average value of DL of all drugs present in the DrugBank database was 0.18, we set the evaluation criteria as drug-likeness (DL) ≥ 0.18.

### Target prediction of bioactive components in DML by the WES model

The WES model, which is known as the drug targeting model, is a calculation model for the fast and accurate prediction of targets and the binding relationship between targets and drugs. It was developed on the basis of similarity ensemble analysis (SEA) by further optimization. The process of target prediction using this model is briefly described. First, the highly relevant physicochemical structural properties and pharmacological properties of drugs were determined through the CDK fingerprint matrix and dragon descriptor matrix under the similarity ensemble framework of the model. Then, the statistical test method was used to establish a matrix for the key characteristics of the ligand set of the protein, and the score of the dependency relationship between the drug and the target was obtained by weighting the similarity of the drug ensemble. Finally, the standardized Z score was integrated by the Bayesian network to obtain the results of target prediction.

### Network construction and results analysis

TCM is a very complex system containing hundreds of different ingredients. The research paradigm of Western medicine of “one target, one drug” may not be applicable to TCM research. Therefore, it is necessary to shift from the current “one target, one drug” mode to a new “network target, multiple components” mode. To solve these problems, we can systematically and comprehensively reveal the relationship between drugs and target proteins by constructing a drug-target network and a target-disease network. The construction of the network helps us identify the protein targets of each compound in the Chinese medicine and understand the mechanisms by which the drugs treat diseases.

The active ingredient-target network and target-disease network were constructed and visualized by using Cytoscape, which is a network visualization and analysis software. Its core function is to provide a basic function layout and query network and form a visual network according to the combination of basic data. In these graphical networks, the compounds, proteins, or pathways are expressed as nodes, whereas the compound-target or target-pathway interactions are expressed as edges. The relationship between the active compound in DML, corresponding targets and related diseases was analysed by the visual network, which helped us obtain more accurate and in-depth insight into the complex mechanism by which DML can treat cardiovascular disease.

### Pharmacological verification of the network analysis

#### Materials

The total flavonoid extract of *D. moldevica* (TFDM) was prepared by the Xinjiang Uygur Autonomous Region Institute of Medicine (batch number: 20180412, 58.4%). The following reagents were used: DMEM (Dulbecco’s modified Eagle’s medium) low glucose medium and 0.25% pancreatin EDTA (ethylenediaminetetraacetic acid) (HyClone, US); DMEM (Dulbecco’s modified Eagle’s medium) glucose-free medium and foetal bovine serum (Gibco, US); cell proliferation and cytotoxicity detection kits (cell counting kit-8, CCK-8) and broad-spectrum phosphatase inhibitors (Boster Bio, US); RIPA (radioimmunoprecipitation assay) lysis solution (strong), PMSF (phenyl methyl sulfonyl fluoride), malondialdehyde (MDA) detection kits and 2′,7′-dichlorofluorescin diacetate (DCFH-DA) (Beijing Solarbio Science & Technology Co., Ltd., China); lactate dehydrogenase (LDH) release detection kits and SDS‒PAGE gel preparation kits (Beyotime Biotechnology); superoxide dismutase (SOD) (Nanjing Jiancheng Institute of Bioengineering, China); BCA protein quantification kits (Thermo Fisher Pierce, US); primary antibodies against P38MAPK, p-P38MAPK, ERK1/2, p-ERK1/2, BaX, BCl-2, (CST Company, US), NOX-4 and PGC-1α (Abcam, US); GAPDH antibodies, horseradish enzyme-labelled goat anti-mouse IgG, and horseradish enzyme-labelled goat anti-rabbit IgG (Zhongshan Jinqiao Company, China) and; MGC, (Mitsubishi Gas Chemical Company. Inc., Japan).

#### Cell culture and OGD/R injury model

The rat cardiomyoblast cell line (H9c2) was obtained from Procell Life Science & Technology Co., Ltd. H9c2 cells were cultured in DMEM low-glucose medium containing 10% foetal bovine serum in 5% CO_2_ at 37 ℃. When cell confluence reached 85%-90%, 0.25% trypsin was added, and the cells were passaged once every 2–3 days.

H9c2 cells (1 × 10^4^ cells/well) were inoculated in 96-well plates with 6 replicate wells and randomly divided into a normal control group (control group) and a hypoxia/reoxygenation model group (model I—IV group). After the cells adhered to the wells, each well was gently washed twice with PBS. The control group was cultured with normal medium. The model group was cultured with glucose-free medium and placed in an anaerobic incubated for 3, 6, 9 and 12 h. Then, the 96-well plates were removed, and the cell viability of each group was detected with CCK-8 reagent. After the hypoxia time required in this experiment was determined, the cells were deprived of oxygen-glucose for 2 h in DMEM low-glucose culture medium containing 10% FBS to induce hypoxia. The cell viability of each group was detected by CCK-8 assays. Finally, the appropriate time for oxygen/glucose deprivation and reperfusion was selected to establish an OGD/R injury model. This model was used to simulate the pathological changes of myocardial ischaemia‒reperfusion injury and related diseases.

#### Cell culture and treatments

H9c2 cells (1 × 10^4^ cells/well) were inoculated in 96-well plates with 6 replicate wells and randomly divided into a normal control group (control group), OGD/R injury model group and TFDM administration groups (25, 50 and 100 μg·mL^−1^). H9c2 cells were cultured in an incubator with 5% CO_2_ at 37 ℃ for 8 h to 10 h. After the cells grew to 60% confluence, each group was pretreated with different concentrations of TFDM for 12 h and then exposed to hypoxia/glucose deprivation for 9 h and reoxygenation/glucose for 2 h to construct the OGD/R injury model.

#### Cell viability assay

H9c2 cells (1 × 10^4^ cells/well) were inoculated in 96-well plates with 6 replicate wells. After being grouped and treated as described in the Cell Culture and Treatment Section, cell viability was measured according to the instructions of the CCK-8 kit. The absorbance at 450 nm was recorded, and the viability of H9c2 cells was calculated with the following formula: cell viability (%) = (OD _experimental group_-OD _blank group_)/(OD _control group_ -OD _blank group_) × 100%.

#### Determination of lactate dehydrogenase levels

After the cells were incubated with different concentrations of TFDM, the culture supernatants of each group were collected, and lactate dehydrogenase (LDH) leakage was determined using a commercial LDH detection kit according to the manufacturer’s instructions.

#### Determination of superoxide dismutase activity and malonaldehyde levels

Whole-cell lysates of H9c2 cells were prepared using RIPA lysis buffer. After the cell lysates were collected, the activities of SOD and MDA were determined with relevant detection kits according to the manufacturer’s directions.

#### Measurement of ROS

ROS generation was evaluated by adding 2’,7’-dichlorofluorescein diacetate (DCFH-DA) to cell culture plates containing 1 × 10^4^ cells/well. The cells were grouped and treated as described in the Cell Culture and Treatment Section. After the cells were incubated, the fluorescence intensity was measured at wavelengths of 488 nm (excitation) and 525 nm (emission) by using a fluorescence microscope.

#### Western blot analysis

H9c2 cells in the logarithmic phase were seeded in 6-well plates and cultured in an incubator with 5% CO_2_ at 37 ℃. After being grouped and treated as described in the Cell Culture and Treatment Section, the cells were washed twice with precooled PBS for Western blot analysis. In brief, H9c2 cells were lysed in RIPA buffer (containing 1% PMSF and 1% phosphatase inhibitor) for 20 min on ice. Subsequently, the lysates were centrifuged at 12,000 rpm for 10 min at 4 °C. The total protein concentration was determined by a BCA protein assay kit. Equal amounts of protein were separated using SDS-PAGE and then electrophoretically transferred onto PVDF membranes. The membranes were blocked with 5% BSA in TBST for 2 h at room temperature and then incubated with primary antibodies at 4 °C overnight. After being washed with TBST buffer three times, the membranes were incubated with HRP-conjugated IgG secondary antibodies for 2 h at room temperature. Immunoreactivity was determined using an advanced ECL kit and visualized using a chemiluminescence imaging system.

### Statistical analysis

Statistical analysis was performed with GraphPad Prism 8.0 software. All data are expressed as the mean ± standard deviation (SD). Differences among multiple groups were evaluated using one-way analysis of variance (ANOVA). The difference between the means was considered statistically significant at *P* < 0.05.

## Results

### Screening the active ingredients of DML

The active ingredients of DML were identified by a pharmacological (TCMSP) analysis platform and literature analysis. All active ingredients were prioritized by predicting their absorption, distribution, metabolism, and excretion (ADME) characteristics with the following criteria: OB ≥ 20% and DL ≥ 0.18. To ensure the accuracy and completeness of the data, a few molecules with biological activity according to the literature were included as candidate active molecules, although they did not meet the screening criteria. Finally, 27 compounds of DML were selected as candidate active molecules, and the results are shown in Table 1.

**Table 1:** Chemical information of 27 DML compounds

NO	Compound	OB	DL	Degree	Structure
mol1	Kaempferol	43.98	0.24	21	
mol2	Quercetin	1.36	0.28	45	
mol3	Diosmetin	38.43	0.27	14	
mol4	Acacetin	43.76	0.24	10	
mol5	Apigenin	46.27	0.21	25	
mol6	Luteolin	20.22	0.25	22	
mol7	Skrofulein	30.72	0.30	15	
mol8	Salvigenin	17.17	0.33	9	
mol9	Santaflavone	13.55	0.40	6	
mol10	Agastachoside	27.03	0.86	3	
mol11	Tilianin	5.19	0.79	2	
mol12	Rosmarinic acid	1.38	0.35	5	
mol13	Diosmetin-7- neohesperidin	11.56	0.70	6	
mol14	Acacetin-7- neohesperidin	35.64	0.74	5	
mol15	Luteolin -7- neohesperidin	3.84	0.73	7	
mol16	Luteolin-7-glucoside	14.07	0.78	5	
mol17	Apigenin -7-glucoside	5.39	0.74	4	
mol18	Luteolin-7- O-β-D- Glucuronide	4.44	0.80	4	
mol19	Apigenin-7-O-β-D-glucuronide	8.82	0.77	4	
mol20	Geraniol-7-O-β-D-glucuronide	16.33	0.83	7	
mol21	Acacidin-7-O-β-D-glucuronide	23.80	0.81	3	
mol22	7,4'-dihydroxyflavone	55.60	0.18	15	
mol23	Agastin	27.03	0.86	3	
mol24	Acacetin-7-o-glucosid-6ʺ-i-malonylester	31.41	0.80	5	
mol25	Acacetin-7-o-β-D- (6 "-o-malonyl) -glucoside	31.41	0.80	5	
mol26	Dracocephaloside A	27.61	0.88	4	
mol27	Acacetin-6ʺ-glucuronide	23.80	0.81	3	

### Compound-target interaction network

**Fig. 1:**
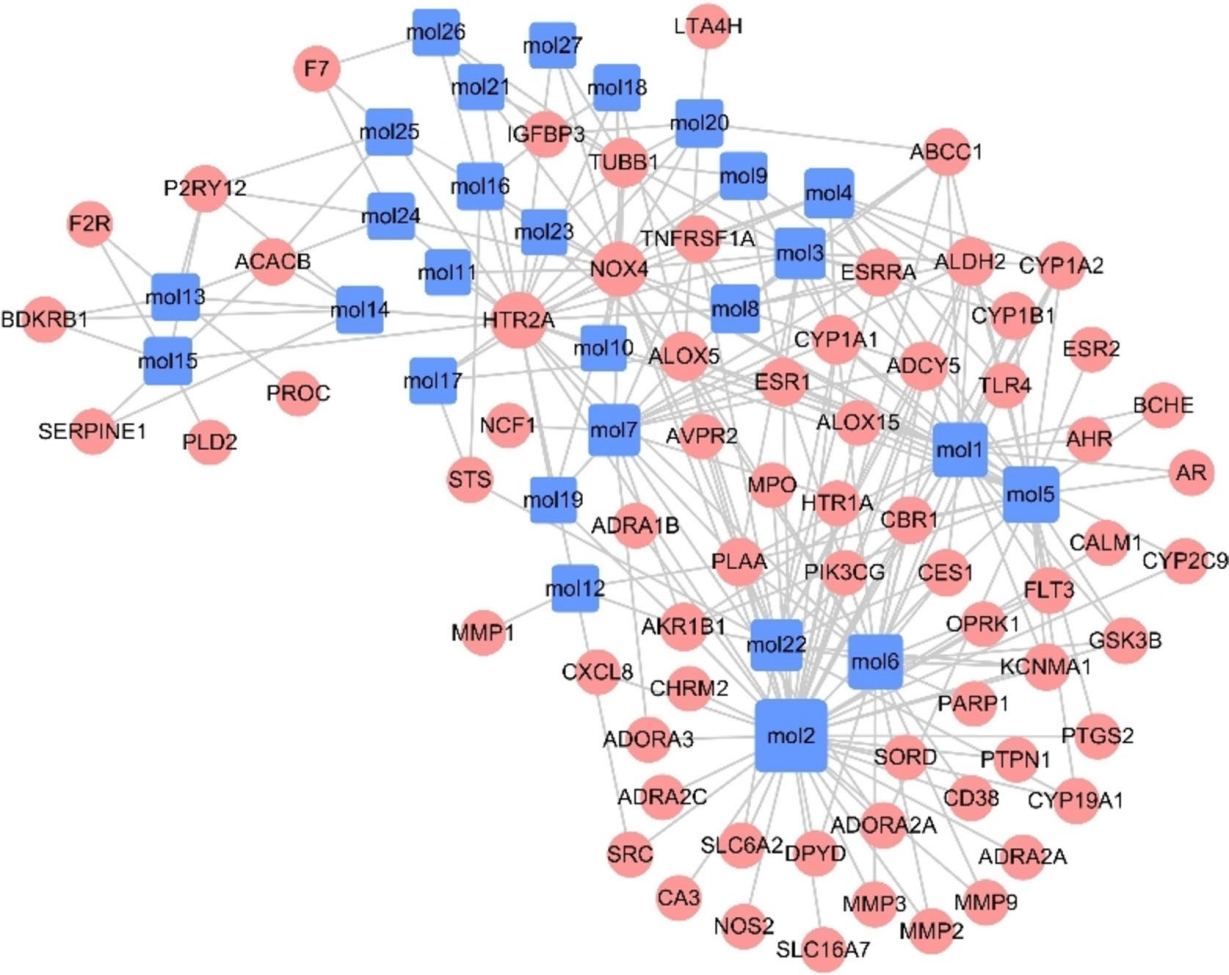
The compound-target network of DML. The purple nodes represent candidate active compounds, and the red nodes represent potential protein targets. The edges represent the interactions between them

Sixty-eight protein targets related to the 27 candidate bioactive components were screened using structure-based drug screening (SBDS) and ligand-based drug screening (LBDS). The compound-target network was constructed, which included 95 nodes and 257 edges (Fig. 1). Among these potential protein targets, there was one high degree target associated with 22 compounds: NOX-4. Moreover, it has been reported that in many CVDs, including atherosclerosis, pulmonary fibrosis and hypertension, heart failure and ischaemic stroke, the expression of NOX-4 is elevated [12].

### Target GOBP enrichment analysis

**Fig. 2:**
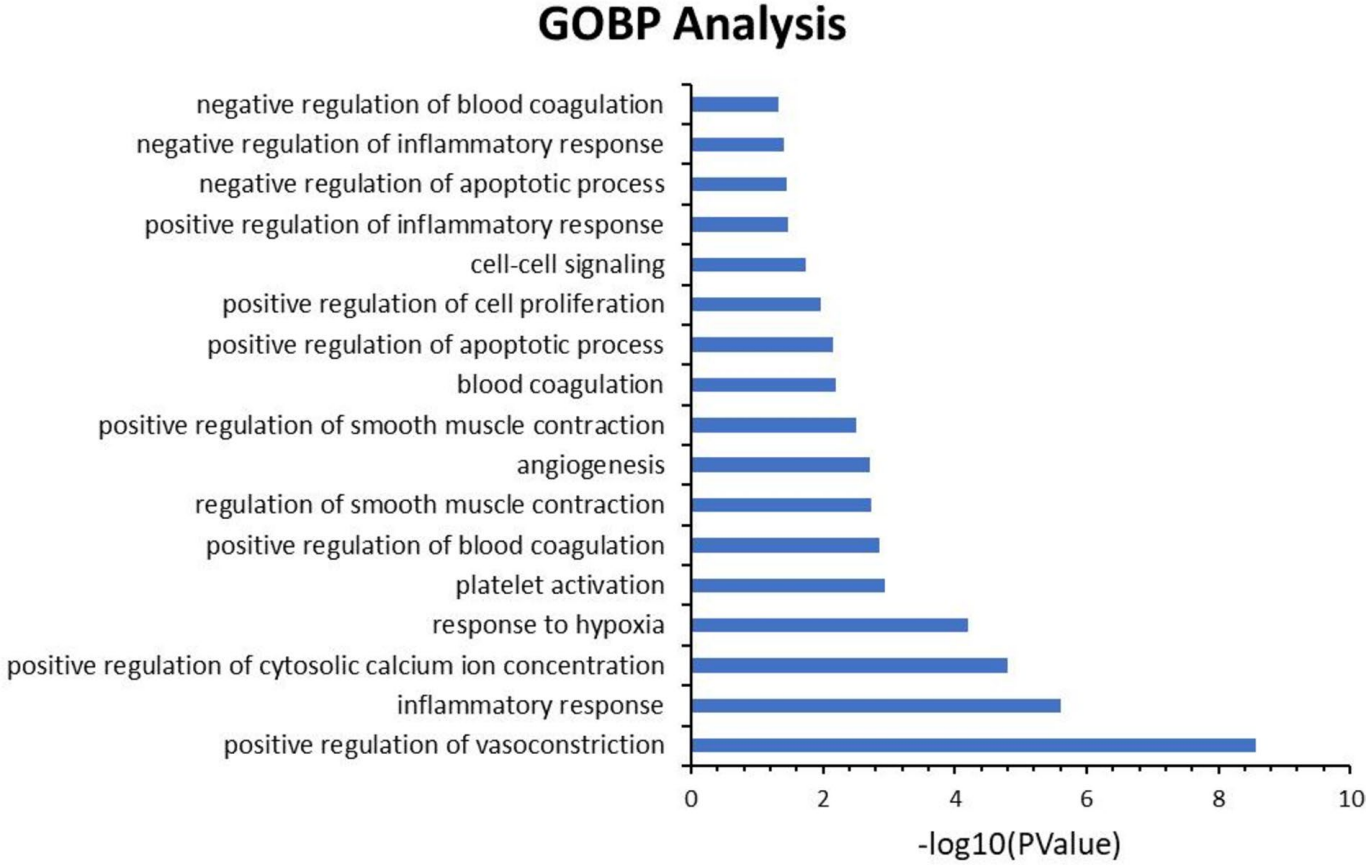
GOBP enrichment analysis of the identified genes encoding proteins targeted by DML in biological processes

GO enrichment analysis of the 68 potential therapeutic targets was performed to identify the relevant biological effects of DML against CVD. The top 17 significantly enriched terms with increased numbers of involved targets in the biological process (BP) are shown in Fig. 2, which indicates that DML may treat cardiovascular disease through the positive regulation of vasoconstriction, the inflammatory response, and cytosolic calcium ion concentration.

### Target-disease interaction network

**Fig. 3:**
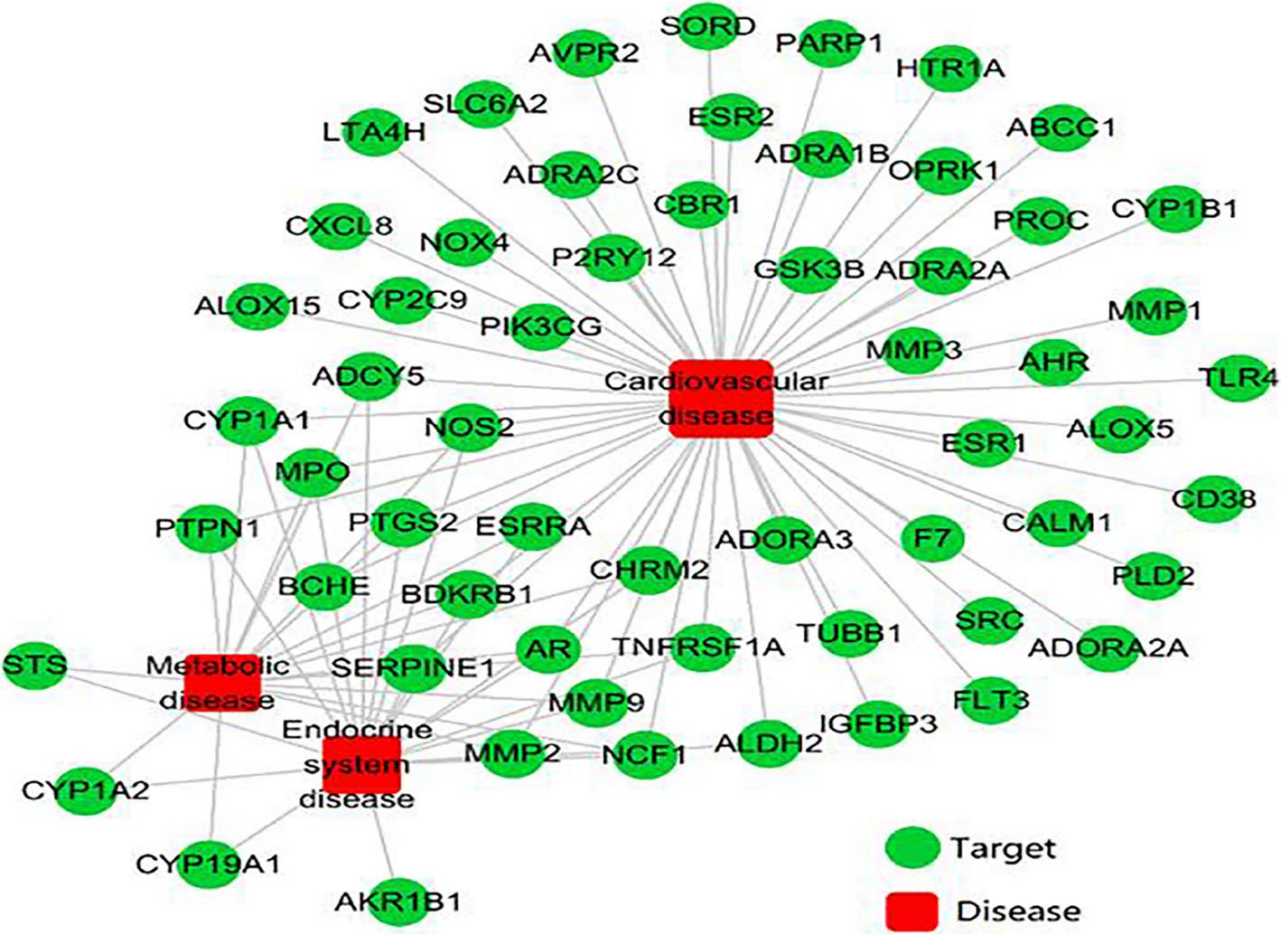
Potential active ingredient target—disease interaction network diagram of the active ingredients of DML

Targets associated with CVD were identified using the DrugBank and GEO databases, and the compound targets and the targets of CVD were mapped by Cytoscape software. There were 59 shared proteins between DML and CVD and 3 types of diseases in the target-disease interaction network (Fig. 3). These targets were associated with multiple active compounds in DML, indicating that the effect of DML on these three diseases involves the synergistic effects of multiple active compounds.

### Construction of new pathways

**Fig. 4:**
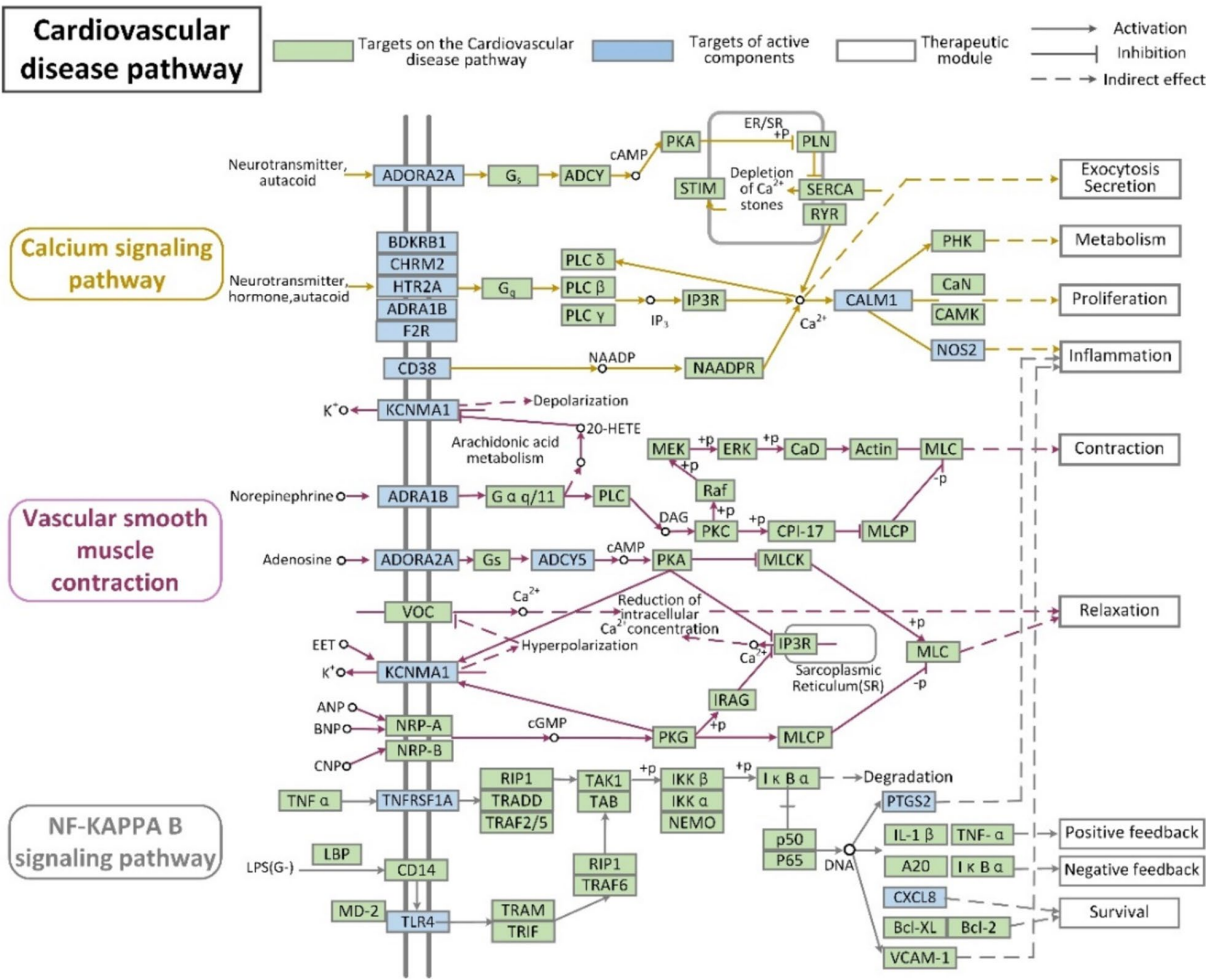
New pathways by which DML can treat CVD (the bioactive components in DML that treat CVD mainly target three signalling pathways and involve nine therapeutic modules)

The treatment of CVD by DML is associated with multiple pathways. As shown in Fig. 4, the calcium signalling pathway was associated with 10 targets and 18 compounds, except molecules 4, 8, 9, 18, 20, 21, 24, 26 and 27; vascular smooth muscle contraction was associated with 4 targets and 9 compounds, including molecules 1–8 and 22; and the NF-kappa B signalling pathway was associated with 4 targets and 8 compounds, including molecules 1–4, 7, 8, 12 and 20. These pathways are divided into several therapeutic modules, such as relaxation, inflammation, and proliferation. For instance, PTGS2 and NOS2 are targeted to inflammatory modules. These target proteins exert inflammatory therapeutic effects by upregulating or downregulating related proteins to counteract related symptoms.

### Experimental validation

#### Effect of TFDM (total flavonoids from *Dracocephalum Moldavica L.*) on the improvement in the viability of OGD/R-stimulated H9c2 Cells

**Fig. 5:**
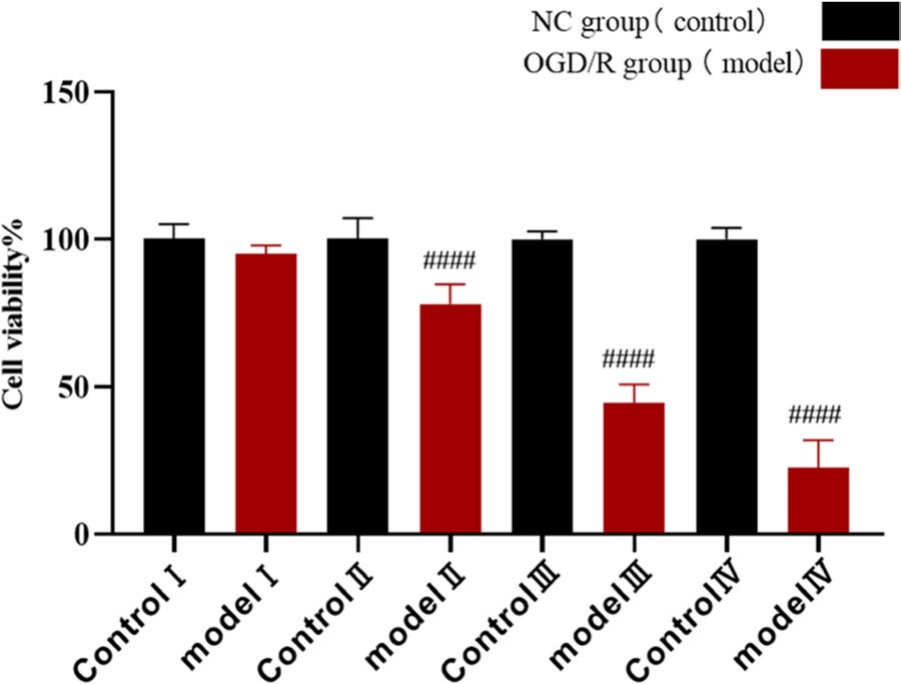
Screening the hypoxia/reoxygenation time in the OGD/R model of H9c2 cells. (*n* = 3, ^####^*P* < 0.0001 versus the control group)

H9c2 cells were subjected to hypoxia, and cell viability was determined after reperfusion for 3, 6, 9, and 12 h. We found that the viability of H9c2 cells decreased with prolonged reperfusion times, as shown in Fig. 5. The viability of H9c2 cells in model I (hypoxia for 3 h and reoxygenation for 2 h), model II (hypoxia for 6 h and reoxygenation for 2 h), model III (hypoxia for 9 h and reoxygenation for 2 h) and model IV (hypoxia for 12 h and reoxygenation for 2 h) was 94.88%, 77.95%, 44.43% and 22.47%, respectively. Therefore, oxygen/glucose deprivation for 9 h and reperfusion for 2 h was the appropriate time to establish an OGD/R injury model.

**Fig. 6:**
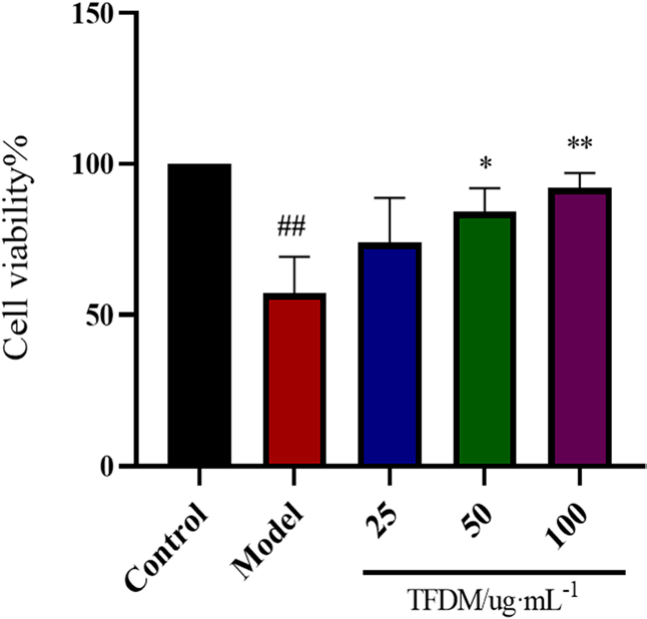
Effect of TFDM on the viability of OGD/R-stimulated H9c2 cells. (*n* = 3, ^##^*P* < 0.01 versus the control group, *P < 0.05 versus the model group, **P < 0.01 versus the model group)

H9c2 cells were pretreated with different concentrations of TFDM (25, 50 and 100 µg/mL) for 12 h. Cell proliferation was measured by a CCK-8 kit. Compared with that in the control group, the proliferation of cells in the OGD/R model group was significantly decreased (*P* < 0.01). Compared with that in the I\R model group, the proliferation of cells in the 50 and 100 µg/mL TFDM groups was significantly increased (*P* < 0.01) (Fig. 6). This finding suggests that TFDM can protect H9c2 cells from oxygen–glucose deprivation/reoxygenation (OGD/R) injury in a dose-dependent manner.

#### Effect of TFDM on the changes in LDH and MDA in H9c2 cells

**Fig. 7:**
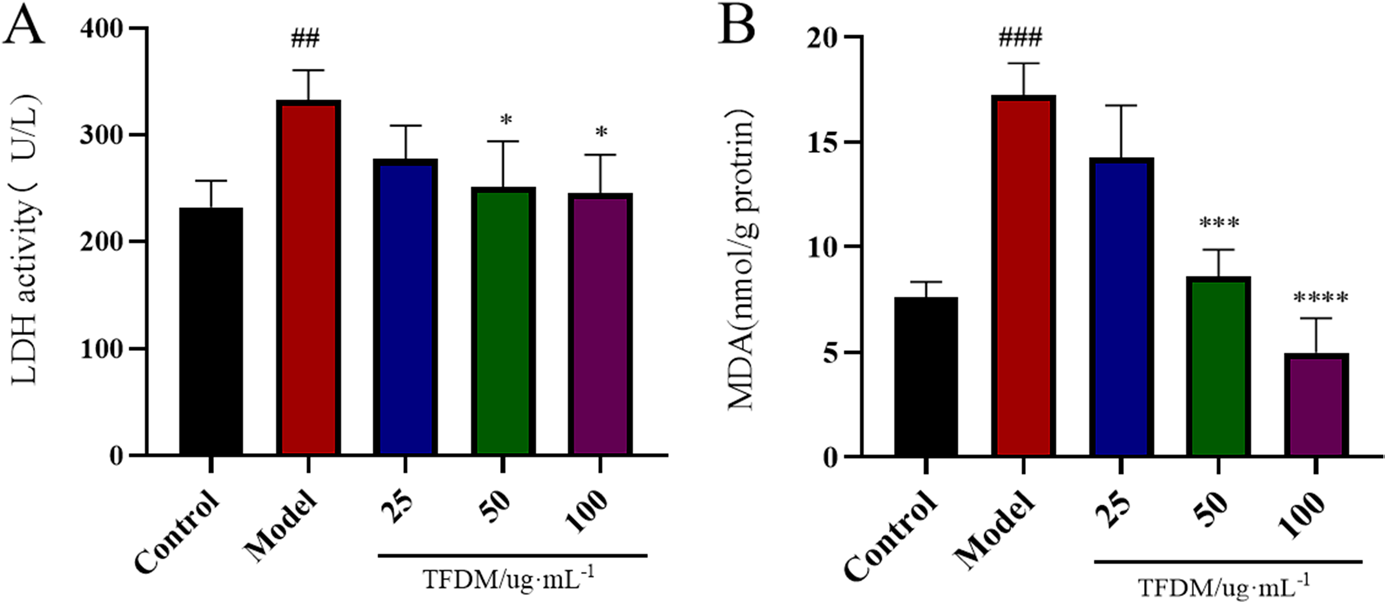
Effect of TFDM on the changes in LDH (**A**) and MDA (**B**) levels in OGD/R-stimulated H9c2 cells. **A** *n* = 3, ^##^*P* < 0.01 versus the control group, *P < 0.05 versus the model group. **B** *n* = 3, ^###^*P* < 0.001 versus the control group, ***P < 0.001 versus the model group, ****P < 0.0001 versus the model group)

To assess the protective effect of TFDM against OGD/R injury in H9c2 cells, the effect of TFDM on the changes in LDH and MDA was evaluated. As shown in Fig. 7, compared with those in the control group, LDH and MDA levels in the OGD/R model group were increased significantly (*P* < 0.01). Compared with those in the OGD/R model group, LDH levels in the 50 and 100 µg/mL TFDM groups were decreased (*P* < 0.01). Compared with those in the OGD/R model group, MDA levels in the 50 and 100 µg/mL TFDM groups were significantly decreased in a dose-dependent manner.

#### Effect of TFDM on the inhibition of oxidative stress in H9c2 cells exposed to OGD/R

**Fig. 8:**
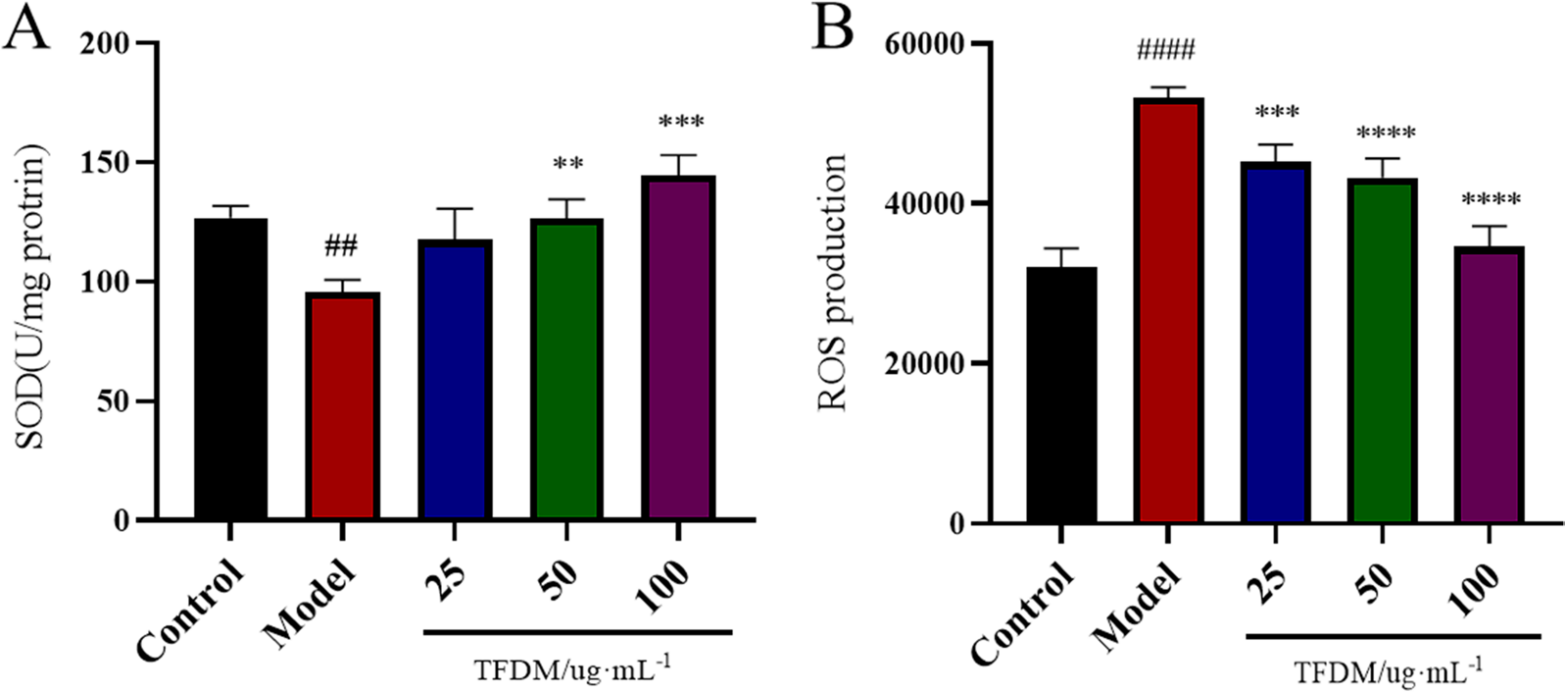
Effect of TFDM on the inhibition of oxidative stress in H9c2 cells exposed to OGD/R. **A** The activity of SOD. *n* = 3, ^####^*P* < 0.0001 versus the control group, **P < 0.01 versus the model group, ****P < 0.0001 versus the model group. **B** The levels of ROS. *n* = 3, ^####^*P* < 0.0001 versus the control group, ***P < 0.001 versus the model group, ****P < 0.0001 versus the model group

The results showed that the OGD/R-induced decrease in SOD activity was blocked by pretreatment with TFDM (Fig. 8A). Moreover, ROS levels were markedly increased by OGD/R conditions compared with those in the control group (*P* < 0.0001), whereas compared with that in the OGD/R group, ROS production in the TFDM group was significantly decreased (Fig. 8B).

#### Effect of TFDM on apoptosis in H9c2 cells exposed to OGD/R injury

**Fig. 9:**
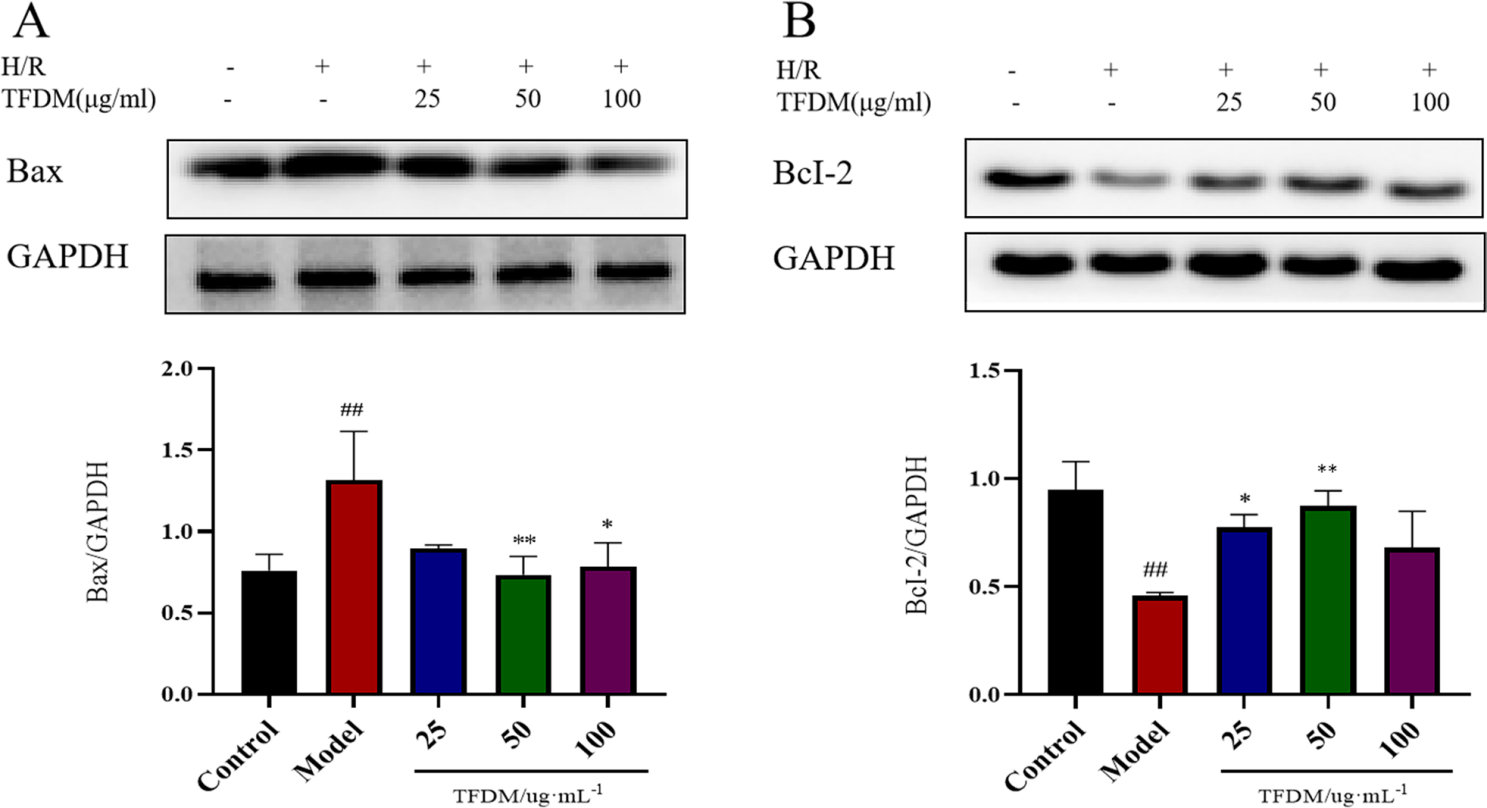
TFDM pretreatment inhibited apoptosis in H9c2 cells after OGD/R stimulation. **A** Western blot analysis of the expression levels and quantification of Bax. *n* = 3, ^##^*P* < 0.01 versus the control group, **P < 0.01 versus the model group, *P < 0.05 versus the model group. **B** Expression levels and quantification analysis of Bcl-2 were measured by western blotting. *n* = 3, ^##^*P* < 0.01 versus the control group, **P < 0.01 versus the model group, *P < 0.05 versus the model group

Next, we verified the effect of TFDM on apoptosis in H9c2 cells exposed to OGD/R injury. The protein expression levels of Bax and Bcl-2 were assessed using western blotting. As shown in Fig. 9A, B, OGD/R caused a significant increase in Bax expression and a decrease in Bcl-2 expression in H9c2 cells. The OGD/R-induced changes in the expression levels of Bax and Bcl-2 were prevented by TFDM.

#### Effects of TFDM on the expression of NOX-4, PGC-1α and other related proteins

**Fig. 10:**
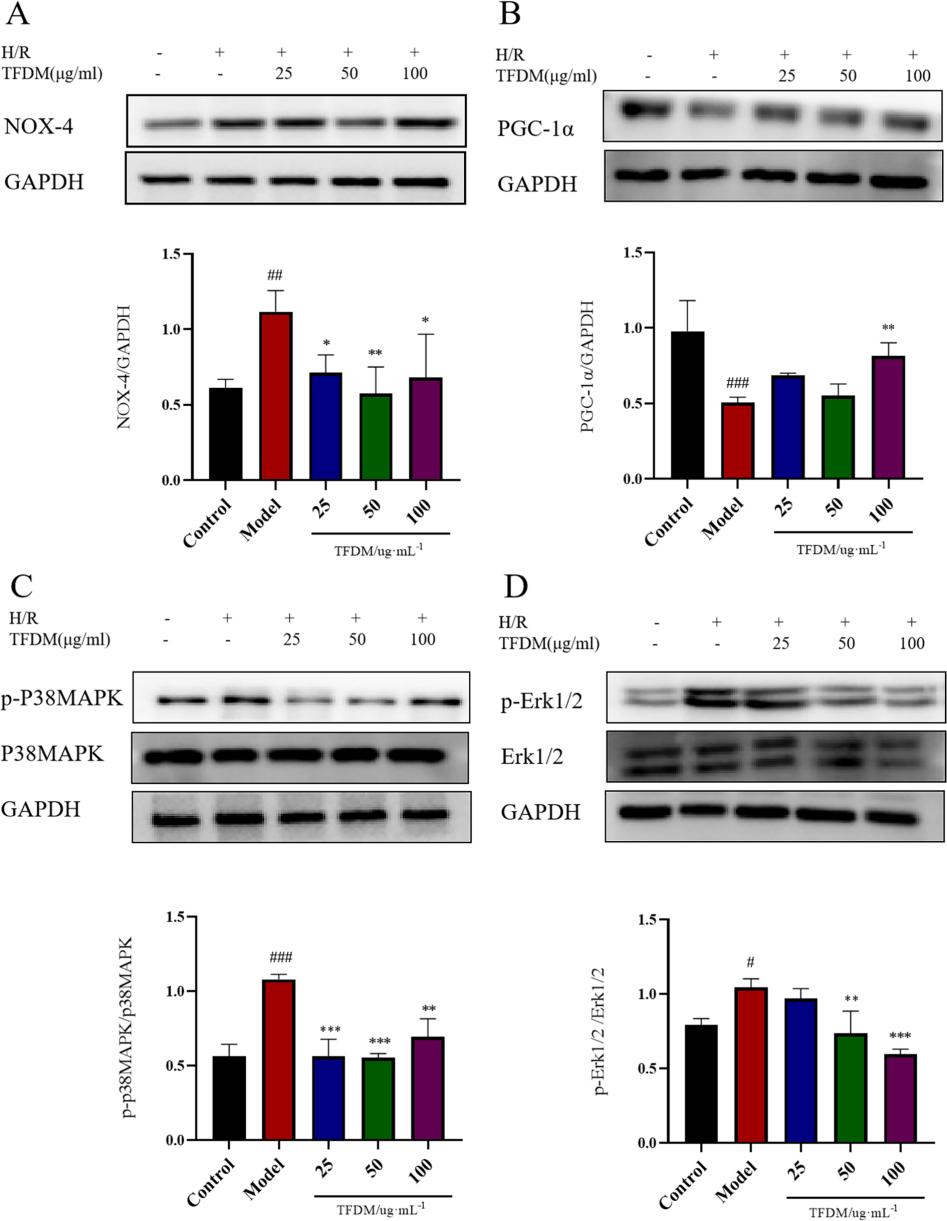
Effects of TFDM on the expression of NOX-4, PGC-1α, p-P38 MAPK/P38 MAPK and p-Erk1/2/Erk1/2. **A** Western blotting was performed to detect the expression levels of NOX-4. *n* = 3, ^##^*P* < 0.01 versus the control group, **P < 0.01 versus the model group, *P < 0.05 versus the model group. **B** Western blotting was performed to detect the expression levels of PGC-1α. *n* = 3, ^####^*P* < 0.0001 versus the control group, ****P < 0.0001 versus the model group, *P < 0.05 versus the model group. **C** Western blotting was performed to detect the expression levels of p-P38 MAPK and P38 MAPK. *n* = 3, ^##^*P* < 0.01 versus the control group, **P < 0.01 versus the model group, *P < 0.05 versus the model group. **D** Western blotting was performed to detect the expression levels of p-Erk1/2 and Erk1/2. *n* = 3, ^#^*P* < 0.05 versus the control group, ***P < 0.001 versus the model group, **P < 0.01 versus the model group

The protein expression levels of NOX-4, PGC-1α, p-P38 MAPK, P38 MAPK, p-Erk1/2 and Erk1/2 were assessed using western blotting. As shown in Fig. 10A, C and D, OGD/R caused a significant increase in NOX-4 expression and the ratios of p-P38 MAPK/P38 MAPK and p-Erk1/2/Erk1/2. Compared with that in the model group, the expression of NOX-4 in the TFDM treatment group was significantly decreased. Similarly, the ratios of p-P38 MAPK/P38 MAPK and p-Erk1/2/Erk1/2 gradually decreased in a dose-dependent manner. In contrast, as shown in Fig. 10B, compared with that in the control group, the expression of PGC-1α in the model group was significantly decreased, and TFDM treatment caused a significant increase in PGC-1α expression.

## Discussion

Myocardial ischaemia‒reperfusion injury seriously restricts the success of treating patients with cardiovascular diseases [12, 13]. A variety of pathophysiological processes, such as oxidative stress [14], mitochondrial dysfunction [15], calcium overload [16], cell inflammation [17], and apoptosis [18], are involved in myocardial OGD/R injury. Therefore, this study focused on the cardioprotective effect against myocardial OGD/R injury to reveal the mechanism by which DML affects cardiovascular disease.

Through a network pharmacology approach, 27 active compounds in DML that can be absorbed into the blood were found. There were 59 potential targets associated with these 27 compounds in the prevention of cardiovascular disease. Among these potential protein targets, NOX-4 [19–21] was a high-degree target associated with 22 compounds that were found high levels in TFDM. TFDM is the main effective component of DML, and TFDM has a variety of physiological activities, such as reducing blood lipids and inhibiting platelet aggregation, coagulation and thrombosis. Furthermore, according to the theory of serum pharmacochemistry, only the components in the blood can exert their effects, and flavonoids account for 6 of the 8 components of DML absorbed in the blood based on a previous study by our group [22]. Therefore, in subsequent pharmacological experiments, we used TFDM to verify the network pharmacology results.

At the onset of reperfusion, several studies have shown that a fast and marked increase in ROS generation occurs in postischaemic tissues [23–25]. SOD plays an important role in oxidation, and as an antioxidant [26], it can effectively prevent tissue damage through oxidation. In our study, we found that TFDM attenuated the overproduction of ROS and reduction in SOD activity induced by OGD/R. Furthermore, TFDM reduced LDH and MDA production. Our findings suggest that the increase in antioxidant activity and the inhibition of the peroxidation of free radicals in the myocardium are involved in the mechanism by which TFDM protects against myocardial OGD/R injury.

Although multiple sources of ROS have been identified, evidence suggests that NADPH oxidases (NOXs) are major contributors to oxidant generation during hypoxia-reoxygenation in different organs [27]. NOXs have been proven to be the main sources of ROS under various pathological conditions, including MIRI [28, 29]. In other words, NOX isoforms play important roles in mediating oxidative stress and myocardial injury after OGD/R, and suppressing NOX activity can effectively prevent the excessive generation of ROS. NOX-4 is the most abundantly expressed isoform of the NOX family and is an important enzymatic source of ROS and a crucial regulator of redox signalling. In cardiomyocytes, NOX4 is mainly expressed in mitochondria [30]. NOX4 may be a molecular target of MIRI. Given that most of the 27 active compounds in DML interact with NOX-4, we focused on the expression of NOX-4 in OGD/R injury and the changes in its expression after TFDM treatment. In our experiment, OGD/R significantly increased NOX-4 expression, and the expression of NOX-4 in the TFDM treatment group decreased significantly. Matsushima et al. showed a decrease in myocardial damage following OGD/R in NOX-2- and NOX-4-deficient mice [31]. In this study, the authors used cardiac-specific NOX-4-knockout mice. All knockout mice exhibited a reduction in ROS production and the attenuation of infarct size after OGD/R, suggesting that NOX-4 in cardiomyocytes plays a dominant role in mediating OGD/R injury. It has also been shown that [30, 31] the downregulation of NOX-4 can reduce oxidative stress by inhibiting mitochondrial dysfunction. Our previous research confirmed that the main component of TFDM, tilianin, can protect mitochondria from damage and reduce the release of inflammatory factors, ultimately protecting myocardial cells from programmed necrosis. In this study, the network pharmacology results indicated that NOX-4 was one of the potential targets of tilianin. Therefore, it is possible that tilianin protects mitochondrial function by targeting NOX-4, thereby protecting H9c2 cells against damage by hypoxia-reoxygenation (H/R) injury.

In addition, the MAPK pathway has a close relationship with ROS production [32, 33]. MAPK signalling, including p38, ERK1/2 and JNK, plays multiple roles in cell death, inflammation and oxidative stress [19, 34, 35]. Activation of the MAPK pathway has been implicated in OGD/R injury, including cardiac OGD/R injury [36, 37]. Therefore, we investigated the MAPK signalling pathway. Compared with those in the model group, the ratios of p-P38 MAPK/P38 MAPK and p-Erk1/2/Erk1/2 in the TFDM treatment group decreased in a dose-dependent manner. Thus, we concluded that TFDM inhibits ROS activation by inhibiting NOX-4 activity and then blocks p38 MAPK phosphorylation to protect against myocardial OGD/R injury.

Given the network pharmacology results, we hypothesize that the protective effect of TFDM on cell viability may be related to the antiapoptotic effect of TFDM during IR injury. Pharmacological experiments in vitro showed that TFDM decreased Bcl-2 protein expression and increased Bax protein expression. In addition, Li Chuang et al. showed that TFDM could reduce the degree of myocardial OGD/R injury by activating the AMPK/SIRT1/PGC-1α signal transduction pathway. PGC-1α, which is a downstream protein of AMPK, is involved in oxidative stress, energy metabolism, apoptosis and autophagy [38, 39]. In our study, Western blot analysis confirmed that compared with that in the control group, the expression of PGC-1α in the model group was significantly decreased, and TFDM treatment caused a significant increase in PGC-1α expression. Thus, our research showed that TFDM could reduce apoptosis in H9c2 cells exposed to IR injury through the Bcl-2/Bax and AMPK/SIRT1/PGC-1α signalling pathways. Therefore, through network pharmacology, we effectively explored the multiple components, targets, and pathways of TFDM in the treatment of CVD.

This study focused on the bioactive components in DML. Through network pharmacology, the main therapeutic targets and pathways of DML in the treatment of cardiovascular diseases were analysed, and an “active ingredients-targets-pathways-diseases” network was constructed. Moreover, some potential key targets and pathways were verified by pharmacological experiments in vitro. The results showed TFDM was the main active ingredient in DML and could exert therapeutic effects on cardiovascular diseases through the NOX-4/ROS/p38 MAPK, Bcl-2/Bax and AMPK/SIRT1/PGC-1α signalling pathways. Due to the numerous targets and signalling pathways involved, we were unable to verify them one by one. In the future, we may choose key compound molecules in TFDM and conduct confirmatory research on targets and signalling pathways. Overall, this systematic study provides a basis for pharmacological studies to further explore the mechanisms of TCM.

### Supplementary Information

**Additional file 1.**

## Data Availability

The datasets generated during the current study are available from the corresponding author upon reasonable request.

## Abbreviations

DML: Dracocephalum moldavica L
CVD: Cardiovascular disease
OGD/R: Oxygen–glucose deprivation/reoxygenation
MIRI: Myocardial ischaemic reperfusion injury
TFDM: Total flavonoids from Dracocephalum Moldavica L
H9c2: Rat cardiomyoblast line (H9c2)
TCMSP: Traditional Chinese Medicine System Pharmacology
OB: Oral bioavailability
DL: Drug likeness
Caco-2: Caco-2 permeability
t1/2: Half-life
BBB: Blood‒brain barrier
ACE: Angiotensin converting enzyme
IHD: Ischaemic heart disease
LMIC: Low- and middle-income countries
TCM: Traditional Chinese medicine
ADME: Absorption, distribution, metabolism, excretion
SEA: Similarity ensemble analysis
DCFH-DA: Dichlorofluorescein diacetate
MDA: Malondialdehyde
LDH: Lactate dehydrogenase
SOD: Superoxide dismutase
CCK-8: Cell proliferation and cytotoxicity detection kit
ROS: Reactive oxygen species
BP: Biological process
LBDS: Ligand-based drug screening
SBDS: Structure-based drug screening
SD: Standard deviation
NOX-4: Recombinant nicotinamide adenine dinucleotide phosphate oxidase 4
PGC-1α: Peroxisome proliferators-activated receptor γ coactivator l alpha
BAX: Anti-Bax antibody
p-P38 MAPK: Phospho-p38 MAPK (Tyr323)
p-Erk1/2: Phospho-Erk1/2 antibody
Bcl-2: Bcl-2 (phospho Thr56) antibody
GAPDH: Mouse anti-GAPDH monoclonal antibody
JNK: C-Jun N-terminal kinase
PMSF: Phenyl methyl sulfonyl fluoride
MGC: AnaeroPackTM -Anaero
