# Supplementary material for: A network pharmacology approach to decipher the mechanism of total flavonoids from Dracocephalum Moldavica L. in the treatment of cardiovascular diseases
Source: BMC Complement Med Ther. 2024 Jan 2;24:15. doi: 10.1186/s12906-023-04316-x (PMC10759627; doi:10.1186/s12906-023-04316-x)

Western blot original strips of NOX-4,ERK1/2,p-ERK1/2,PGC-1a, p-P38MAPK ,P38MAPK,Bax,Bcl-2 and GAPDH.

Bax

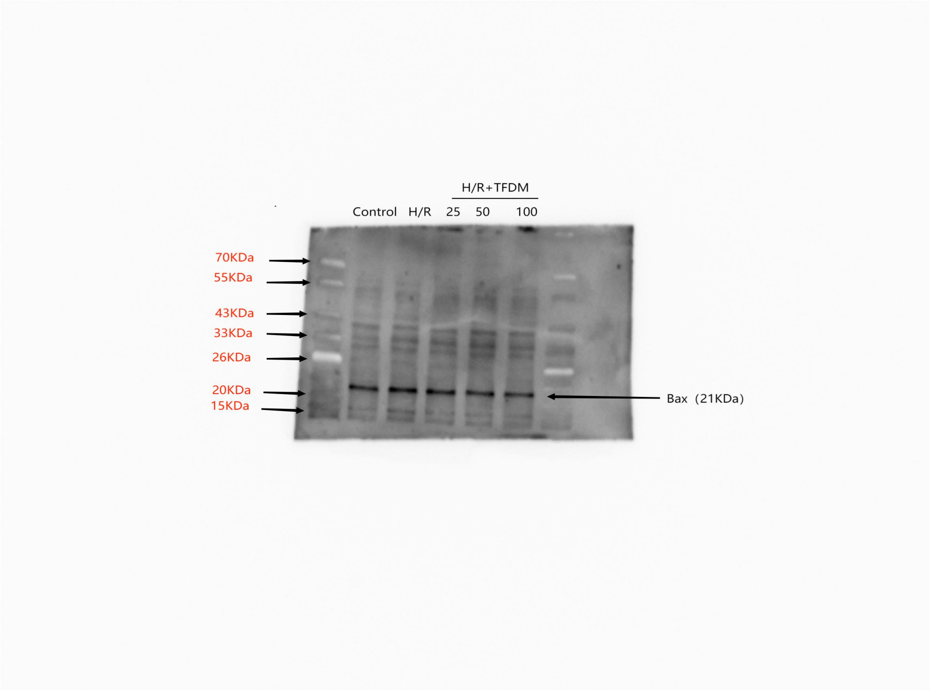

GAPDH1

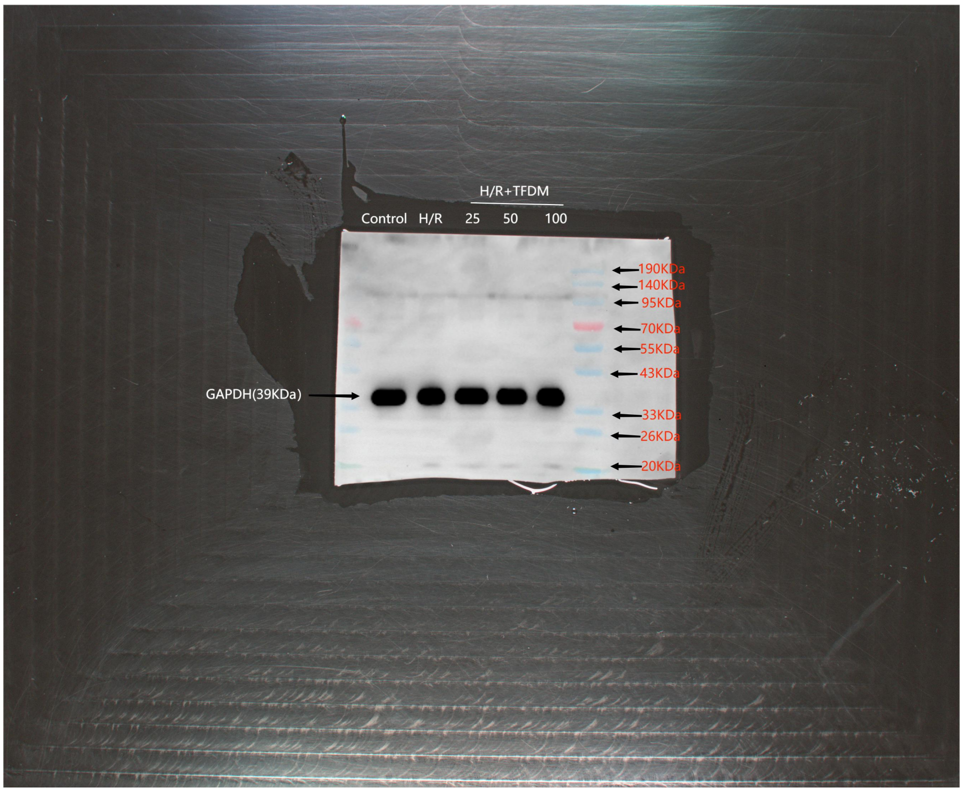

Bcl-2

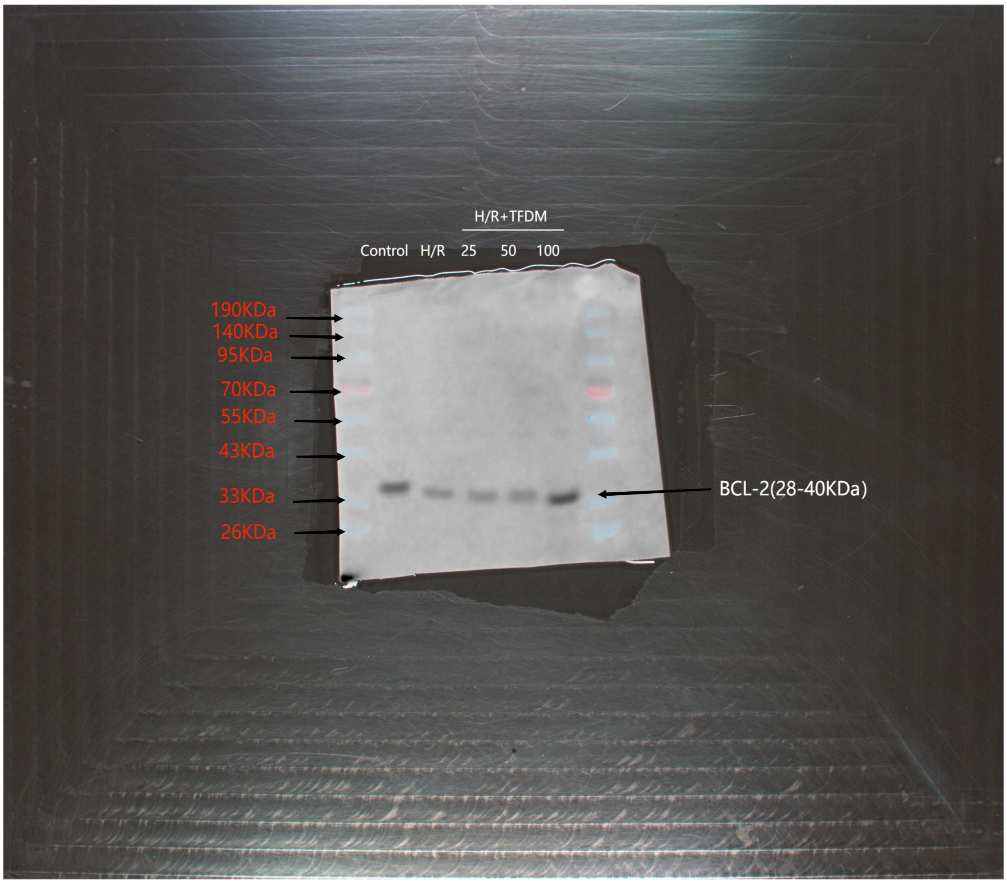
H/R+TFDM
Control
H/R
25
50
100
190KDa
140KDa
95KDa
70KDa
55KDa
43KDa
33KDa
26KDa
BCL-2(28-40KDa)

GAPDH2

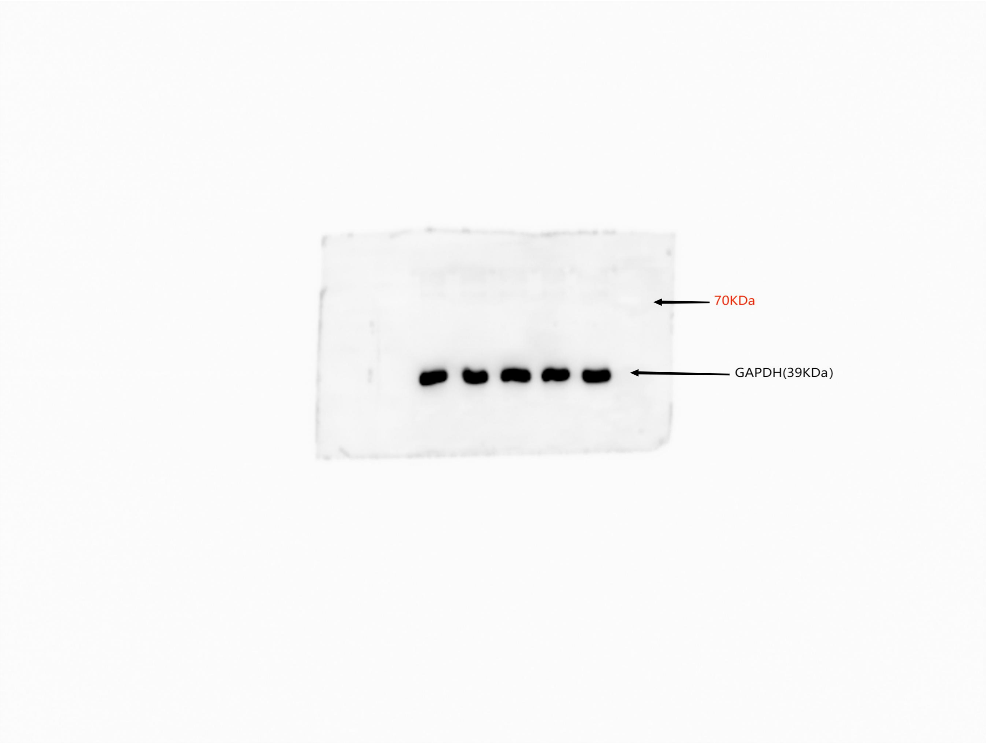
70KDa
GAPDH(39KDa)

NOX-4

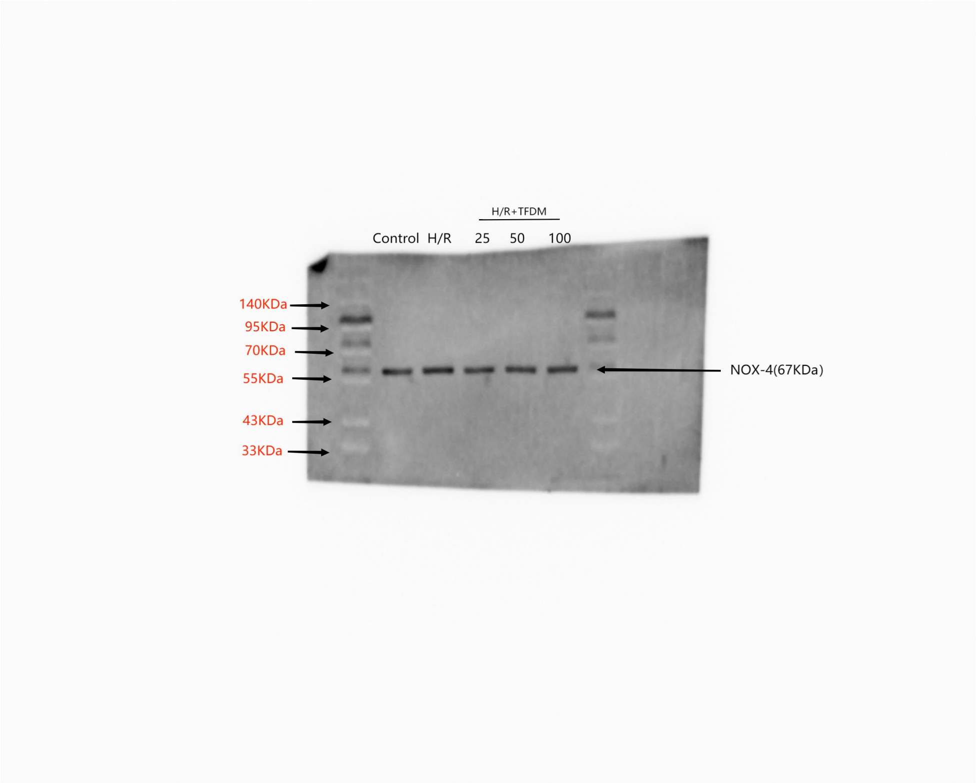
H/R+TFDM
Control H/R 25 50 100
140KDa
95KDa
70KDa
55KDa
43KDa
33KDa
NOX-4(67KDa)

GAPDH3

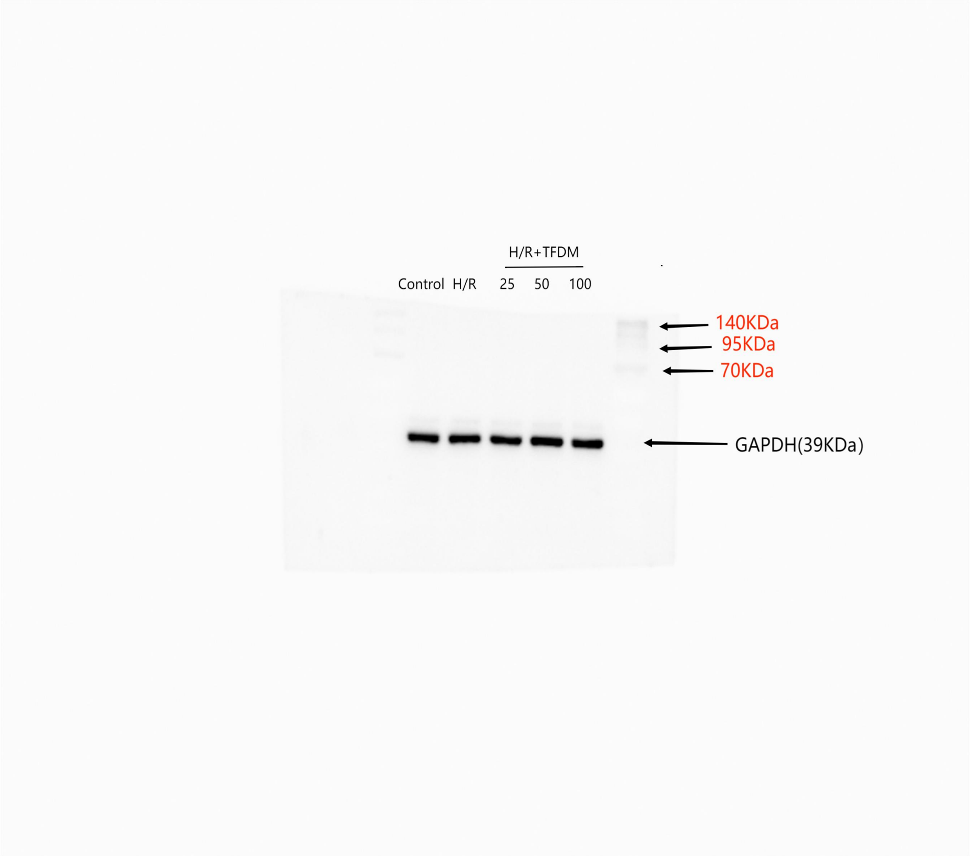
H/R+TFDM
Control H/R 25 50 100
140KDa
95KDa
70KDa
GAPDH(39KDa)

PGC-1a

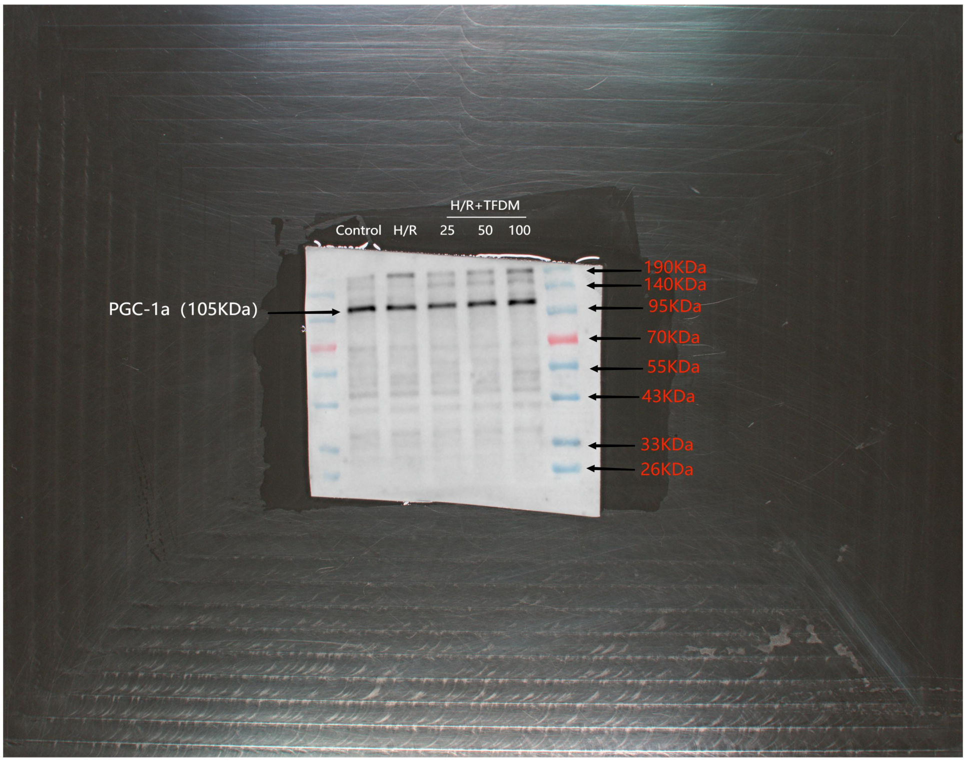

GAPDH3

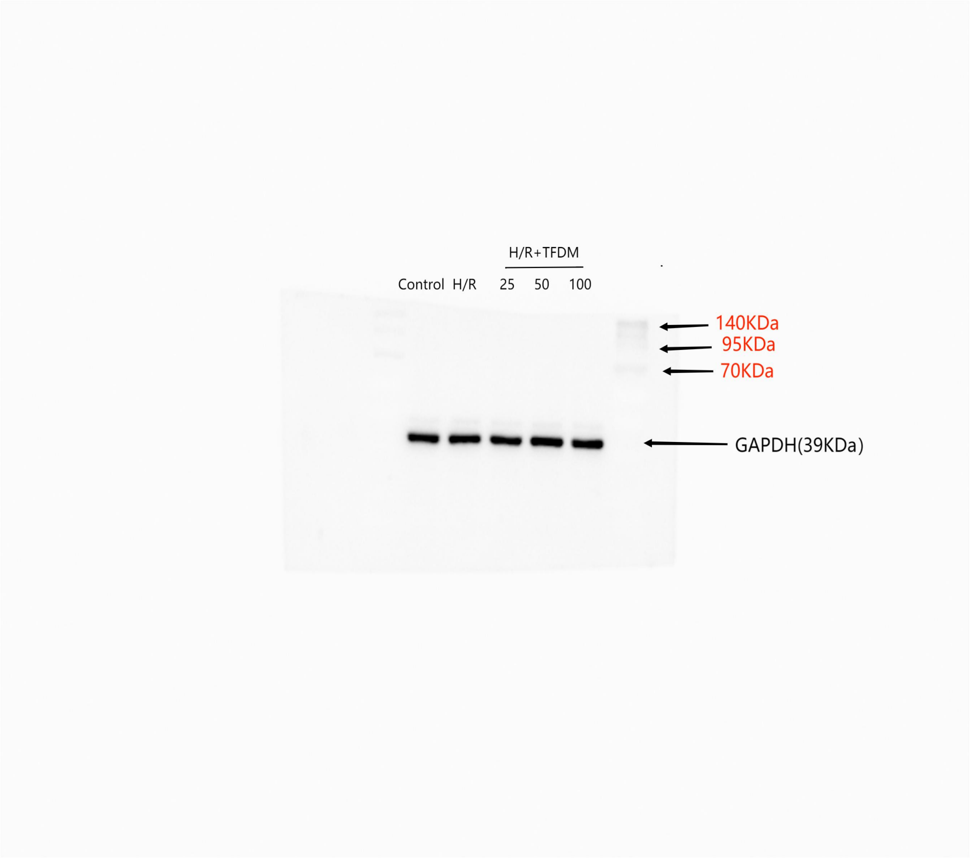

ERK1/2

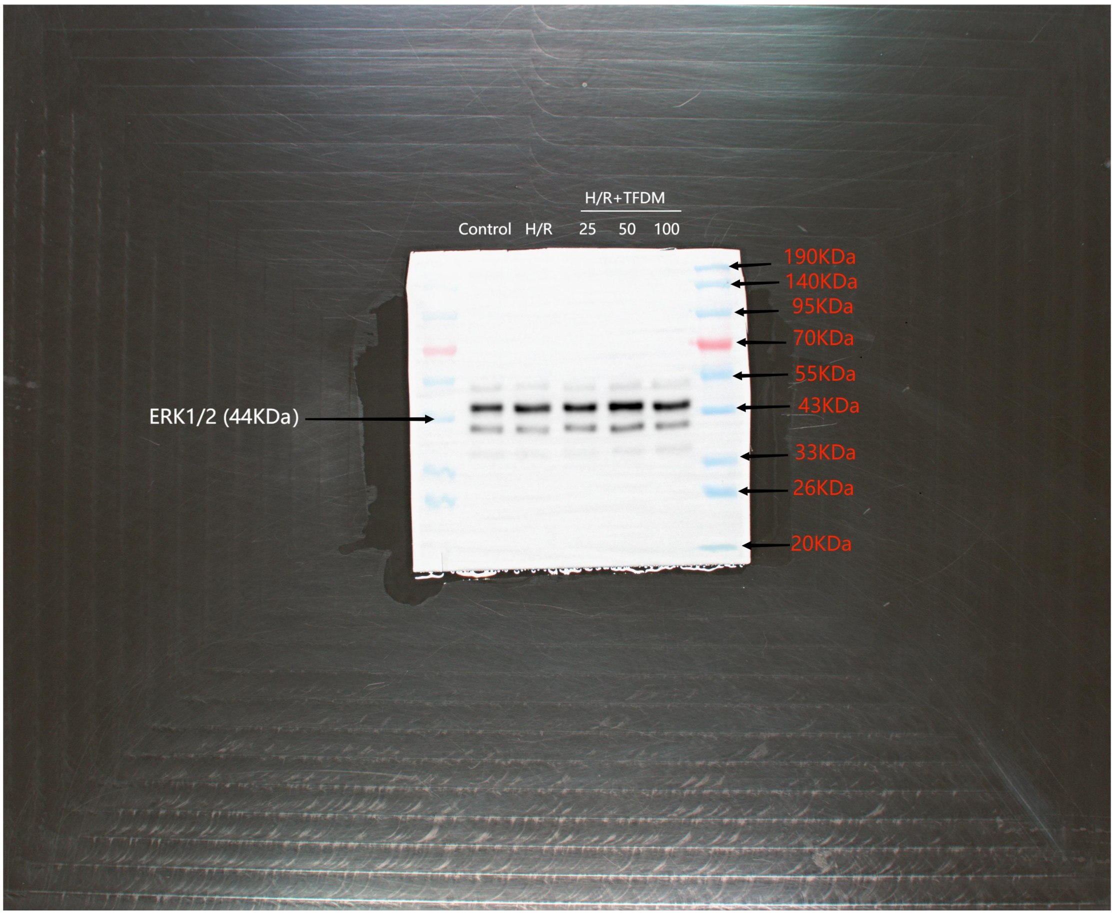

p-ERK1/2

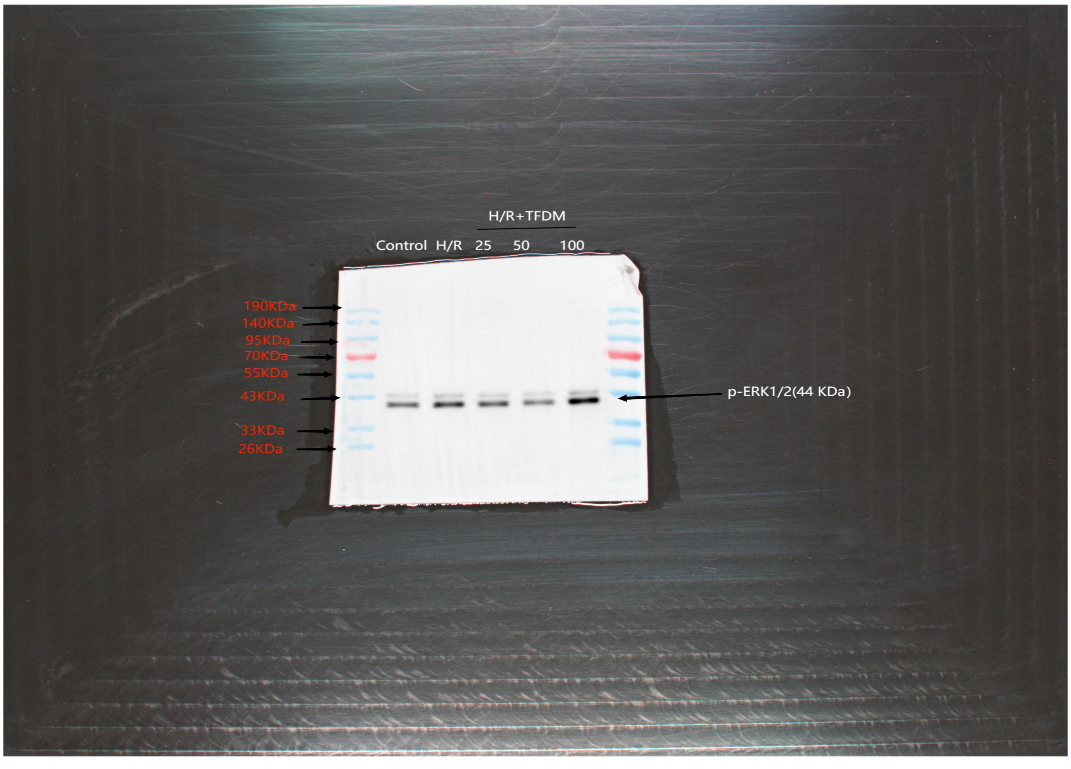

P38MAPK

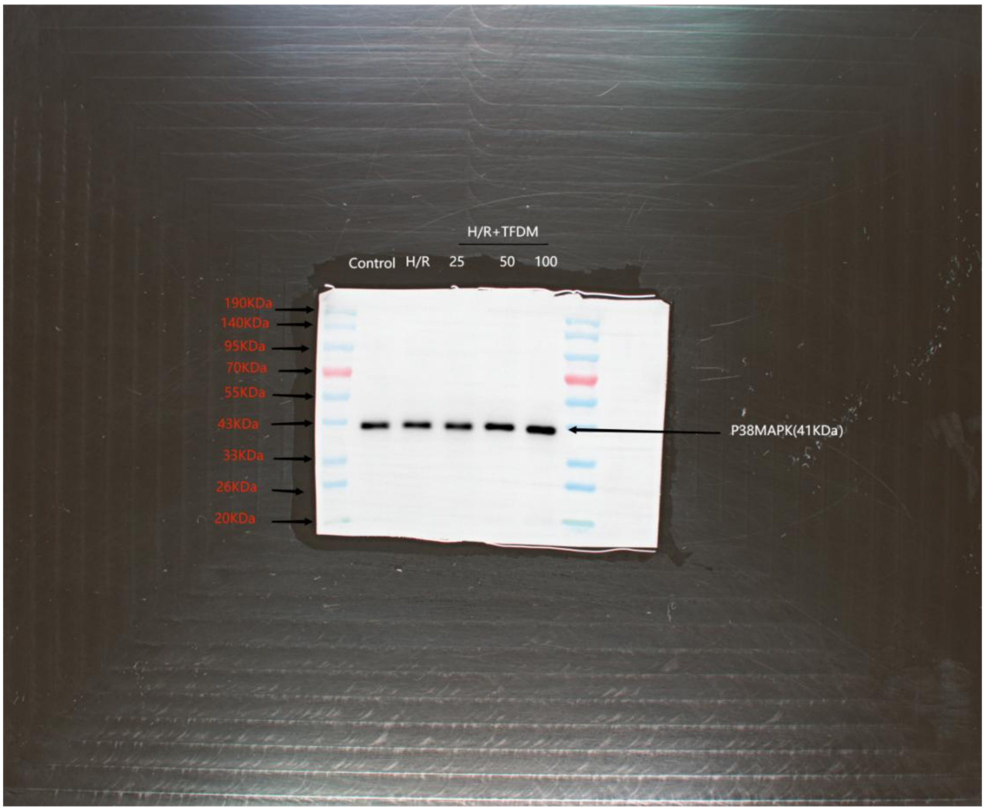

p-P38MAPK

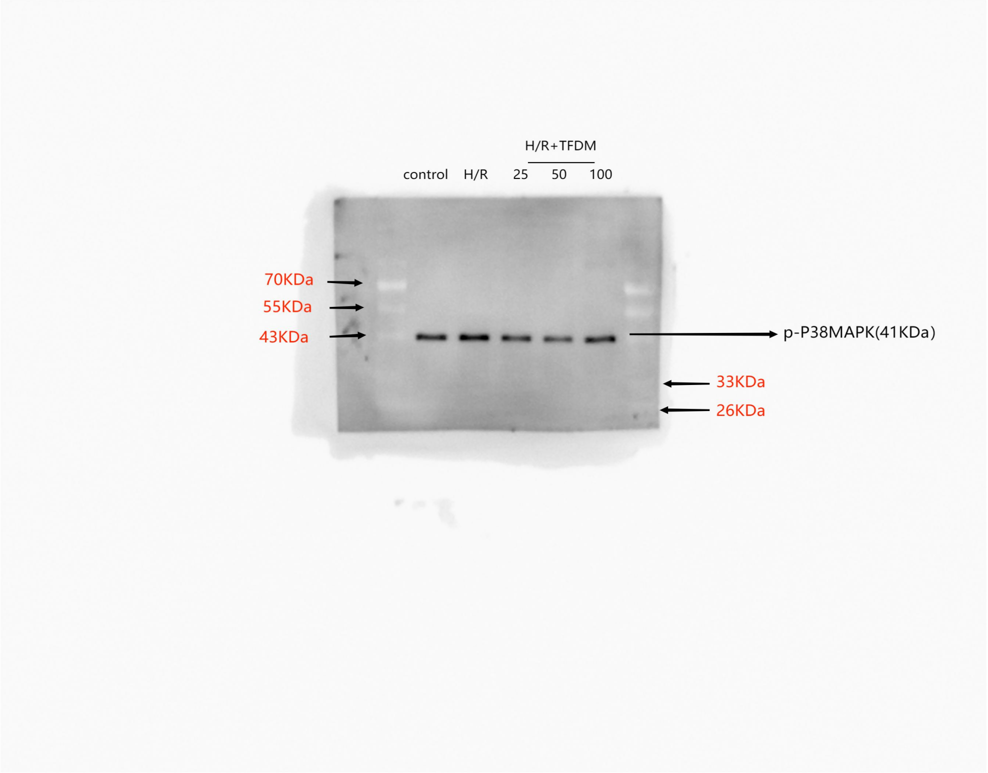

GAPDH

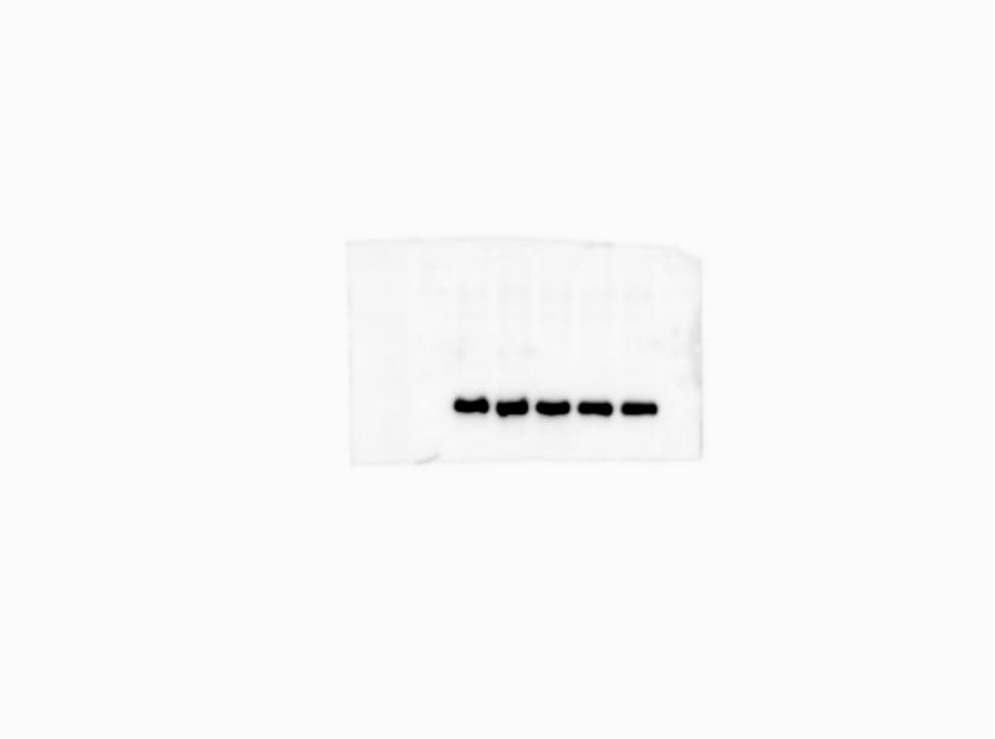

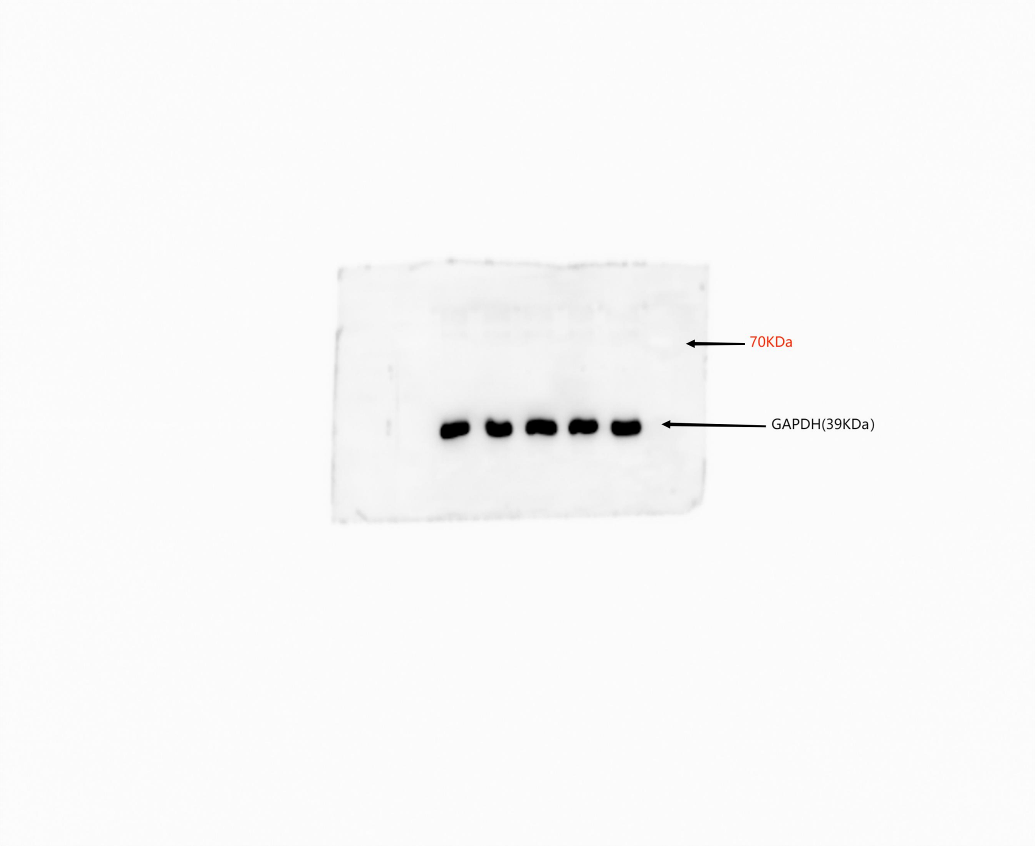

Supplement: Supplementary file 1 — Additional file 1. [file 12906_2023_4316_MOESM1_ESM.pdf]
